# Antiproliferative, Antimicrobial and Apoptosis Inducing Effects of Compounds Isolated from *Inula viscosa*

**DOI:** 10.3390/molecules17033291

**Published:** 2012-03-14

**Authors:** Wamidh H. Talib, Musa H. Abu Zarga, Adel M. Mahasneh

**Affiliations:** 1 Department of Clinical Pharmacy and Therapeutics, Applied Science University, Amman, 11931-166, Jordan; 2 Department of Chemistry, University of Jordan, Amman, 11942, Jordan; 3 Department of Biological Sciences, University of Jordan, Amman, 11942, Jordan

**Keywords:** flavonoids, anticancer, antimicrobial, quercetin, hispidulin

## Abstract

The antiproliferative and antimicrobial effects of thirteen compounds isolated from *Inula viscosa* (L.) were tested in this study. The antiproliferative activity was tested against three cell lines using the MTT assay. The microdilution method was used to study the antimicrobial activity against two Gram positive bacteria, two Gram negative bacteria and one fungus. The apoptotic activity was determined using a TUNEL colorimetric assay. Scanning electron microscopy was used to study the morphological changes in treated cancer cells and bacteria. Antiproliferative activity was observed in four flavonoids (nepetin, 3,3′-di-*O*-methylquercetin, hispidulin, and 3-*O*-methylquercetin). 3,3′-di-*O*-Methylquercetin and 3-*O*-methylquercetin showed selective antiproliferative activity against MCF-7 cells, with IC_50_ values of 10.11 and 11.23 µg/mL, respectively. Both compounds exert their antiproliferative effect by inducing apoptosis as indicted by the presence of DNA fragmentation, nuclear condensation, and formation of apoptotic bodies in treated cancer cells. The antimicrobial effect of *Inula viscosa* were also noticed in 3,3′-di-*O*-methylquercetin and 3-*O*-methyquercetin that inhibited *Bacillus cereus* at MIC of 62.5 and 125 µg/mL, respectively. *Salmonella typhimurium* was inhibited by both compounds at MIC of 125 µg/mL. 3,3′-di-*O*-Methylquercetin induced damage in bacterial cell walls and cytoplasmic membranes. Methylated quercetins isolated from *Inula viscosa* have improved anticancer and antimicrobial properties compared with other flavonoids and are promising as potential anticancer and antimicrobial agents.

## 1. Introduction

Treatment of cancer and microbial infections have drawn the attention and interest of researchers due to their great impact on the population’s health. Two to 3% of deaths recorded worldwide annually arise from different types of cancer [1]. On the other hand and due to the uncontrolled use of antibiotics, bacteria and fungi have evolved numerous mechanisms to evade old and new antimicrobial agents [2]. In the quest for new therapeutics, plants were and still are considered as one of the main sources of biologically active materials. It has been estimated that about 50% of the prescription products in Europe and USA are originated from natural products, including plants or their derivatives [3,4]. During the last 20 years, apoptosis (programmed cell death) has been extensively studied as an ideal method to kill cancer cells and chemical agents that induce apoptosis have been reported to be promising tools to control malignant cancer [5]. *Inula viscosa* (L.) Aiton (*Compositae*) (common local name: Taioon) is a perennial plant distributed in different regions of the Mediterranean Basin [6]. In traditional medicine, *Inula viscosa* has many uses, including anti-inflammatory [7], anthelmintic, lung disorders [8], antipyretic, antiseptic, and antiphlogistic activities [9,10] in addition to treating gastroduodenal disorders [11]. Crude extracts prepared from different parts of *Inula viscosa* exhibit antifungal [12], antioxidant [13], antiulcerogenic [14] and anthelmintic [15] properties and prevent zygote implantation [16]. In previous studies we reported the potent antiproliferative [17] and antimicrobial [18] activities of an *Inula viscosa* methanol extract. Chemical analysis showed that *Inula viscosa* contains many biologically active compounds, including flavonoids and terpenoids [9]. Fourteen known and four new compounds were isolated from Jordanian *Inula viscosa* [19]. *In vitro* antiproliferative and antimicrobial screening of plant products can provide valuable preliminary data for the potential use of these products to treat cancer and/or microbial infections. Taking into account the use of *Inula viscosa* in traditional medicine, its wide distribution, and the lack of studies that evaluate the biological activity of its pure compounds, the present study was undertaken to evaluate the antiproliferative, antimicrobial and apoptosis induction effects of thirteen compounds isolated from *Inula viscosa*. 

## 2. Results and Discussion

In the present study, thirteen compounds previously isolated and identified from *Inula viscosa* [19] were tested for their antiproliferative, antimicrobial, and apoptosis induction activities. Among the tested compounds, four flavonoids exhibited high antiproliferative activity against different cell lines. These flavonoids are nepetin, 3-*O*-methylquercetin, 3,3′-di-*O*-methylquercetin, and hispidulin. The most potent compound was nepetin, with IC_50_ values of 5.87, 11.33, and 103.54 µg/mL against MCF-7, Hep-2, and Vero cell lines, respectively (Table 1). Our results agree with previous studies that reported high antiproliferative activity of nepetin isolated from *Eupatorium ballotaefolium* HBK. (Asteraceae) [20] and *Inula britannica* L. (Asteraceae) [21]. Hispidulin also showed significant (*p* < 0.05) antiproliferative activity against MCF-7 and Hep-2 cell lines with IC_50_ values of 10.35 and 19.50 µg/mL, respectively. However, its antiproliferative activity against Vero cell line was limited, with an IC_50_ value of 105.48 µg/mL (Table 1). Such activity of hispidulin is in accordance with the previous findings that showed the ability of this small flavonoid to suppress growth of different cancer cell lines including HeLa, MK-1, B16F10, glioblastoma multiforme, and human pancreatic cancer [22,23,24].

**Table 1:** IC_50_ determination of compounds isolated from *Inula viscosa.* Values were reported as the average of three replicates. The antiproliferative effect of the tested compounds was determined by comparing the optical density of the treated cells against the optical density of the control.

Compound	IC_50_ (µg/mL)		
	MCF-7	Hep-2	Vero
2α-Hydroxyilicic acid	>150	>150	>150
Hispidulin	10.35 ± 1.85	19.50 ± 1.06	105.48 ± 2.35
3-*O*-Methylquercetin	11.23 ± 1.93	26.12 ± 2.18	>150
3,3′-di-*O*-Methylquercetin	10.11 ± 1.15	28.01 ± 1.13	>150
Nepetin	5.87 ± 1.36	11.33 ± 0.98	103.54 ± 2.82
Inuviscolide	>150	148.78 ± 1.25	>150
β-*S*itosteryl glucoside	>150	>150	>150
2-Desacetoxyxanthinin	>150	>150	>150
Viscic acid	>150	>150	>150
3-*O*-Acetylpadmatin	>150	>150	>150
Ilicic acid	>150	>150	>150
Xepetin	>150	>150	>150
11α,13-Dihydroinuviscolide	>150	>150	>150
Vincristine sulfate	10.03 ± 1.34	>90	>90

Quercetin is one of the most widely distributed flavonoids in different fruits and vegetables [25]. Many pharmacological effects of quercetin have been documented, including anticancer, anti-inflammatory, antibacterial and muscle relaxing effects [26]. *In vivo* studies showed that quercetin metabolism involves modifications inside the liver, followed by the release of the modified products to the circulation. Such modifications include conversion of quercetin into methylated, sulfated, and glucuronidated metabolites [27]. In the present study, two naturally occurring methylated quercetins, 3-*O*-methylquercetin and 3,3′-di-*O*-methylquercetin, isolated from *Inula viscosa* showed high antiproliferative activity against the MCF-7 cell line, with IC_50_ values of 11.23 and 10.11 µg/mL, respectively. Both methylated compounds were also active against the Hep-2 cell line, with IC_50_ values of 26.12 and 28.01 µg/mL for 3-*O*-methylquercetin and 3,3′-di-*O*-methylquercetin, respectively. On the other hand, the toxicity of these two compounds was limited against Vero cell line, with IC_50_ values above 150 µg/mL for both. The biological effects of flavonoids depend mainly on their chemical structure and relative orientation of different moieties in the molecule [25]. Our results report for the first time the antiproliferative activity of naturally occurring 3,3′-di-*O*-methylquercetin. It seems that the presence of additional methyl group on position 3^'^ did not improve the antiproliferative activity of 3,3′-di-*O*-methylquercetin compared with 3-*O*-methylquercetin.

**Figure 1:**
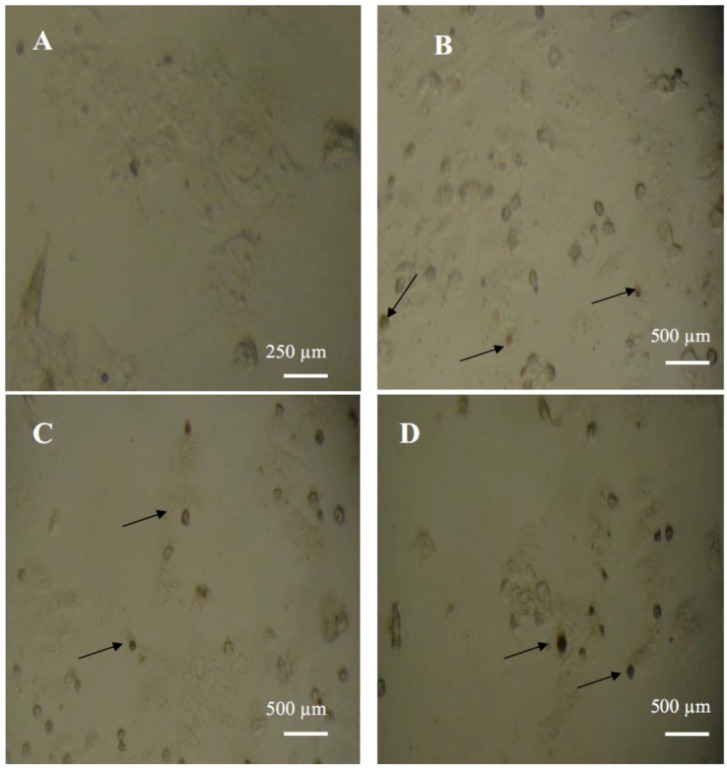
MCF-7 cells assayed by DeadEndTM colorimetric TUNEL system to indicate cell apoptosis. (**A**) Negative control; (**B**) Positive control; (**C**) Cells treated with 15 µg/mL of 3-*O*-methylquercetin; (**D**) Cells treated with 15 µg/mL of 3, 3′-di-*O*-methylquercetin. Arrows show dark stained nuclei which indicate DNA fragmentation and nuclear condensation.

Apoptosis (programmed cell death) is the main form of cell death that is involved in diverse processes ranging from cell development to stress response [28]. Many cancers depend on apoptosis inactivation to survive. Thus, apoptosis induction seems to be one of the effective methods to kill cancer cells [29]. The ability of 3-*O*-methylquercetin and 3,3′-di-*O*-methylquercetin to induce apoptosis in MCF-7 cells were tested using a TUNEL colorimetric assay which detects DNA fragmentation during programmed cell death. Both compounds caused an increase in the number of apoptotic cells with fragmented DNA, compared with untreated MCF-7 cells (Figure 1). 

**Figure 2:**
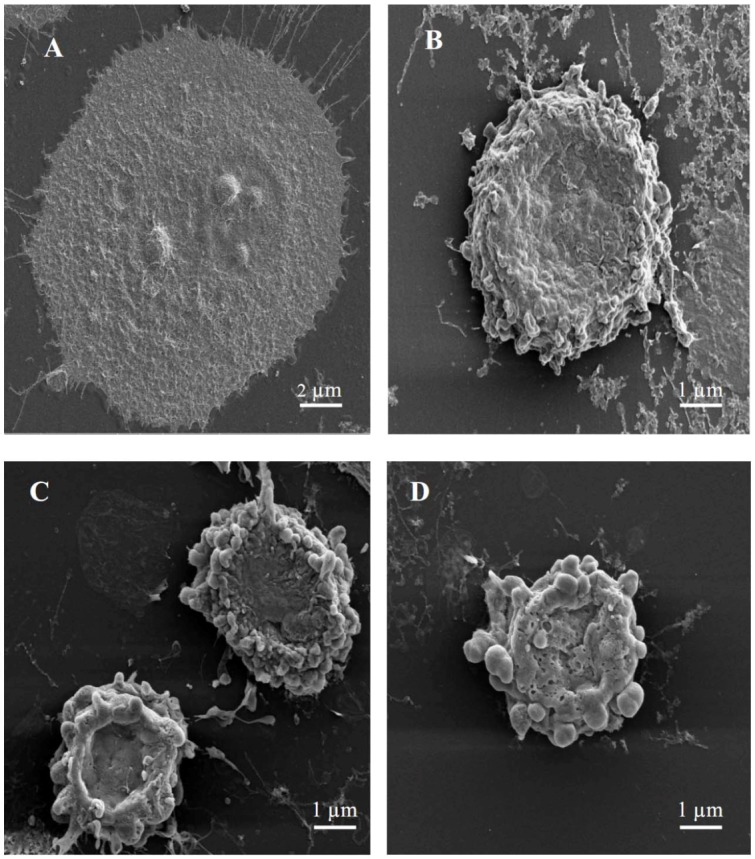
Scanning electron micrographs of MCF-7 cells. (**A**) Untreated cells; (**B**) Cells treated with 40 nM vincristin sulfate; (**C**) Cells treated with 15 µg/mL of 3-*O*-methylquercetin; (**D**) Cells treated with 15 µg/mL of 3,3′-di-*O*-methylquercetin.

In order to gain more insight into cell death, scanning electron microscopy was performed for MCF-7 cells treated with either 3-*O*-methylquercetin or 3,3′-di-*O*-methylquercetin. Cytomorphological alterations corresponding to a typical morphology of apoptosis were detected in MCF-7 cells treated with either 3-*O*-methylquercetin or 3,3′-di-*O*-methylquercetin. These alterations include cell shrinkage, membrane blebbing, loss of contact with neighboring cells, and formation of apoptotic bodies (Figure 2). The presence of partly degraded apoptotic bodies around cells was also detected (Figure 2). On the contrary, untreated cells showed well preserved morphology while cells treated with vincristine (positive control) showed morphological changes similar to those observed in cells treated with either 3-*O*-methylquercetin or 3,3′-di-*O*-methylquercetin (Figure 2).

The second part of this study involved testing the antimicrobial activity of the *Inula viscosa* compounds against different pathogenic microorganisms. All compounds were inactive against *E. coli* and MRSA, except 3,3′-di-*O*-methylquercetin and nepetin, that inhibited MRSA growth at 250 µg/mL. The two flavonoids 3-*O*-methylquercetin and 3,3′-di-*O*-methylquercetin inhibited *Salmonella typhimurium* at 125 µg/mL for both. On the other hand, the MIC of 3,3′-di-*O*-methylquercetin against *Bacillus cereus* was 62.5 µg/mL, while 3-*O*-methylquercetin inhibited the same bacterium at 125 µg/mL. *Candida albicans* was inhibited only by 3-*O*-methylquercetin and 3,3′-di-*O*-methylquercetin at MIC of 250 µg/mL (Table 2). Other compounds exhibited limited or no antimicrobial activity. Structure-activity relationship for antimicrobial activity of flavonoids is greatly dependent on the type and position of substitutions on these compounds [30]. Previous studies reported the antimicrobial activity of quercetin and quercetin-3-*O*-arabinoside against *Bacillus cereus* with MIC values of 350 µg/mL for each [31]. Quercetin-3-*O*-glycoside inhibited *Bacillus cereus* at 50 µg/mL [32]. Our result showed that naturally occurring 3-*O*-methylquercetin and 3,3′-di-*O*-methylquercetin exhibited improved antimicrobial activities compared with some modified quercetin compounds.

**Table 2:** Minimum inhibitory concentration (MIC) in µg/mL of *Inula viscosa* compounds. Microbial species: Methicillin resistant *Staphylococcus aureus* (MRSA); *Bacillus cereus* (B.c); *Escherichia coli* (E.c); *Salmonella typhimurium* (S.t); C*andida albicans* (C.a). ND: not determined. Pure tetracycline, penicillin G, and nystatin were included for comparative purposes.

Compound	Test microorganism				
	MRSA	E.c	S.t	B.c	C.a
2α-Hydroxyilicic acid	>250	>250	>250	>250	>250
Hispidulin	>250	>250	>250	>250	>250
3-*O*-Methylquercetin	>250	>250	125	125	250
3,3′-di-*O*-Methylquercetin	250	>250	125	62.5	250
Nepetin	250	>250	>250	>250	>250
Inuviscolide	>250	>250	>250	>250	>250
β-Sitosteryl glucoside	>250	>250	>250	>250	>250
2-Desacetoxyxanthinin	>250	>250	>250	>250	>250
Viscic acid	>250	>250	>250	>250	>250
3-*O*-Acetylpadmatin	>250	>250	>250	>250	>250
Ilicic acid	>250	>250	>250	>250	>250
Xepetin	>250	>250	>250	>250	>250
11α,13-Dihydroinuviscolide	>250	>250	>250	>250	>250
Tetracycline	125	150	4	2	ND
Penicillin G	>250	125	125	>250	ND
Nystatin	ND	ND	ND	ND	25

**Figure 3:**
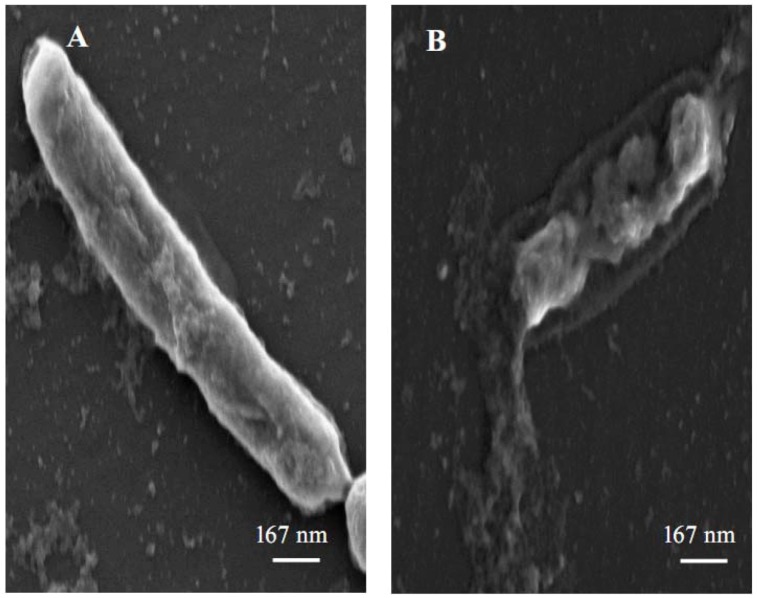
Scanning electron micrograph of Bacillus *cereus* cells. (**A**) Untreated cells; (**B**) Cells treated with 62.5 µg/mL 3,3′-di-*O*-methylquercetin.

For a better understanding of the antimicrobial effect of 3,3′-di-*O*-methylquercetin on *Bacillus cereus*, exponentionally growing bacterial cells were treated with 62.5 µg/mL of 3,3′-di-*O*-methylquercetin for 24 h and were then studied under scanning electron microscope. Damages on both the bacterial cell wall and the cytoplasmic membrane were observed in bacterial cells treated with 3,3′-di-*O*-methylquercetin (Figure 3), while control cells, in the absence of 3,3′-di-*O*-methylquercetin, were intact and have smooth surfacea. Leakage of intracellular constituents was also observed in treated cells. According to previous studies, the loss of cell integrity and membrane permeability is a direct result of the damage of cell wall and cytoplasmic membrane [33,34]. Altering the cell physical structure causes expansion and increase cell membrane fluidity, which cause leakage of vital intracellular components [35]. Many antimicrobial agents exert their effects by induction of leakage of intracellular material [23]. It seems that the antimicrobial activity of 3,3′-di-*O*-methylquercetin involves cell wall and cytoplasmic membrane permeablization that cause the leakage of many vital components from the inside of the cell and lead to cell death.

## 3. Experimental

### 3.1. Plant Material and Extraction Method

*Inula viscosa* was collected from the west of Amman, Jordan. The taxonomic identity of the plant was authenticated by Prof. Dawud EL-Eisawi (Department of Biological Sciences, University of Jordan, Amman, Jordan). Voucher specimens were deposited in the Department of Biological Sciences, University of Jordan, Amman, Jordan. *Inula viscosa* pure compounds were previously isolated and characterized [19]. Briefly, petroleum ether was used to remove fat from the dried plant material followed by ethanol extraction. The crude ethanol extract was subjected to solvent-solvent partitioning between chloroform and water. The dried chloroform extract was also partitioned between *n*-hexane and 10% aqueous methanol. The aqueous methanol extract was chromatographed using Silica gel S (70–230 mesh, Merck) to produce six fractions. A combination of column chromatography (silica gel, 400 mech, Merck) and TLC was used to further purify each fraction [19].

### 3.2. Cell Lines and Culture Conditions

Hep-2 (larynx carcinoma, ATCC: CCL-23), MCF-7 (breast epithelial adenocarcinoma, ATCC: HTB-22), and Vero (African green monkey kidney, ATCC: CCL-81) cell lines were kindly provided by Dr. Mona Hassuneh (Department of Biological Sciences, University of Jordan). Cells were grown in Minimum Essential Medium Eagle (Gibco, Paisley UK) supplemented with 10% heat inactivated fetal bovine serum (Gibco, Paisley UK), 29 μg/mL L-glutamine, and 40 μg/mL gentamicin. Cells were incubated in a humidified atmosphere of 5% CO_2_ at 37 °C.

### 3.3. Microbial Strains

The microbial strains used in this study were *Escherichia coli* (ATCC 10798), *Salmonella typhimurium* (ATCC14028), Methicillin resistant *Staphylococcus aureus* (MRSA, Clinical isolate), *Bacillus cereus* (Toxigenic strain TS), and *Candida albicans* (ATCC 90028). Bacterial strains were stocked onto nutrient agar slant at 4 °C, and *Candida albicans* was stocked onto malt agar slant at 4 °C.

### 3.4. Antiproliferative Activity Assay

The antiproliferative activity of *Inula viscosa* compounds was measured using the MTT (3-(4,5-dimethylthiazol-2-yl)-2,5-diphenyltetrazolium bromide) assay (Promega, Madison, WI, USA). The assay detects the reduction of MTT by mitochondrial dehydrogenase to blue formazan product, which reflects the normal function of mitochondria and cell viability [36]. Exponentially growing cells were washed and seeded at 17,000 cells/well for Hep-2 cell line and 11,000 cells/ well for MCF-7 cell line (in 200 μL of growth medium) in 96 well microplates (Nunc, Roskilde, Denmark). After 24 h incubation, a partial monolayer was formed then the media was removed and 200 μL of the medium containing the compound (initially dissolved in DMSO) were added and re-incubated for 48 h. Then 100 μL of the medium were aspirated and 15 μL of the MTT solution were added to the remaining medium (100 μL) in each well. After 4 h contact with the MTT solution, blue crystals were formed. One hundred μL of the stop solution were added and incubated further for 1 h. 

Reduced MTT was assayed at 550 nm using a microplate reader (Biotek, Winooski, VT, USA). Control groups received the same amount of DMSO (0.1%).Untreated cells were used as a negative control while, cells treated with vincristine sulfate were used as a positive control. Eight concentrations (150, 100, 50, 20, 10, 5, 2, and 1 μg/mL) were prepared from each compound and tested against the three cell lines. IC_50_ values were calculated as the concentrations that show 50% inhibition of proliferation on any tested cell line. Stock solutions of the compounds were dissolved in (DMSO) then diluted with the medium and sterilized using 0.2 μm membrane filters. The toxicity of DMSO was determined and the final dilution of compounds used for treating the cells contained not more than 0.1% (non toxic concentration) DMSO. IC_50_ values were reported as the average of three replicates. The antiproliferative effect of the tested compounds was determined by comparing the optical density of the treated cells against the optical density of the control (cells treated with media containing 0.1% DMSO).

### 3.5. Antimicrobial Assay

The microtiter plate dilution method [37] was used to determine the MIC (minimum inhibitory concentration) of compounds under study. Sterile 96-well microplates (Nunc) were used for the assay. Stock compounds were dissolved in DMSO so that the final concentrations in micro wells were less than 1% DMSO and solvent controls were run at these concentrations. Positive controls of penicillin G and tetracycline (Oxoid, Hampshire, UK) were prepared at the following concentrations 2, 4, 8, 16, 31, 62.5, 125, and 250 μg/mL. 1% DMSO was used as a solvent control. Compounds were diluted to twice the desired initial test concentration (250 µg/mL) with Muller Hinton broth (MHB) (Oxoid). All wells, except the first were filled with MHB (50 μL). Compounds (100 μL) were added to the first well and serial two-fold dilutions were made down to the desired minimum concentration (2 μg/mL). An over-night culture of bacteria suspended in MHB was adjusted to turbidity equal to 0.5 McFarland standard. The plates were inoculated with bacterial suspension (50 μL/well) and incubated at 37 °C for 24 h [18]. Then the turbidity was measured using micro-plate reader (Biotek) at 620 nm wavelength. The same procedure was used to determine the antifungal activity of compounds against *Candida albicans*. The medium used was malt extract broth. The yeast cells were suspended in 0.85% saline, with an optical density equivalent to 0.5 McFarland standard, and diluted 1:100 in malt extract broth to obtain the working concentration [38]. After 24 h incubation with each compound, the turbidity was measured at 530 nm wavelength using a spectrophotometer (Biotek). Percentage inhibition for tested microorganisms was calculated using the following formula:


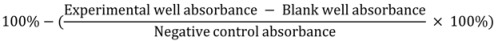


MIC was determined as the lowest concentration of the compound that causes complete inhibition (100%) of each microorganism.

### 3.6. Assessments of Apoptosis in Cell Culture

Apoptosis was detected using terminal deoxynucleotidyl transferase (TdT) mediated-16-deoxyuridine triphosphate (dUTP) Nick-End Labelling (TUNEL) system (Promega, Madison, WI, USA). MCF-7 cells cultured in 24 well plates were treated with 15 µg/mL of either 3-*O*-methylquercetin or 3,3′-di-*O*-methylquercetin for 28 h. The assay was conducted according to the manufacturer's instructions. Briefly, treated cells were fixed using 10% formalin followed by washing with phosphate buffer saline (PBS). Cells were then permeabilized using 0.2% triton X-100. Biotinylated dUTP in rTdT reaction mixture was added to label the fragmented DNA at 37 °C for one hour, followed by blocking endogenous peroxidases using 0.3% hydrogen peroxide. Streptavidin HRP (1:500 in PBS) was added and incubated at room temperature for 30 min. Finally, hydrogen peroxide and chromagen diaminobenzidine were used to visualize nuclei with fragmented DNAs under the light microscope (Novex, Arnhem, Holland). Cells treated with 40 nM vincristine sulfate were used as a positive control while untreated cells were used as a negative control.

### 3.7. Scanning Electron Microscopy

MCF-7 cells were cultured in complete DMEM containing either 15 µg/mL of 3-*O*-methylquercetin or 3,3′-di-*O*-methylquercetin and incubated for 48 h in humidified CO_2_ incubator. Negative control cells were incubated with complete DMEM and positive control cells received complete DMEM containing 40 nM vincristin sulfate. Treated cells were fixed with 3% glutaraldehyde for 1.5 h followed by osmium tetroxide (2% in PBS) for 1 h. After fixation, cells were washed in PBS and sequentially dehydrated using 30%, 70%, and 100% ethanol [39]. Fixed cells were attached to a metal stubs and sputter coated with platinum by using Emitech K550X coating unit (Qourum Technology, West Sussex, UK). The coated specimens were viewed using Inspect F50/FEG scanning electron microscope (FEI, Eindhoven, The Netherlands) at accelerating voltage of 2–5 kV. *Bacillus cereus* cells treated with 3,3′-di-*O*-methylquercetin for 24 h were collected by centrifugation and washed with sodium phosphate buffer. Following that, the samples were fixed in 2.5% glutaraldehyde in 0.1 M phosphate buffer, pH 7.3, at 4 °C overnight, and postfixed in 1% osmium tetroxide in the phosphate buffer for 1 h at room temperature [40]. Bacterial cells were dehydrated with sequential ethanol concentrations (30–100%) and coated for the observations under scanning electron microscope.

### 3.8. Statistical Analyses

The results are presented as means ± SEM of three independent experiments. Statistical differences among compounds were determined by one way ANOVA using Graph Pad Prism 5 (GraphPad Software Inc., San Diego, CA, USA). Differences were considered significant at *p* < 0.05.

## 4. Conclusion

Naturally occurring methylated quercetin isolated from *Inula viscosa* have improved anticancer and antimicrobial properties compared with other flavonoids and are promising as potential anticancer and antimicrobial agents.
